# Association between biomass fuel use and maternal report of child size at birth - an analysis of 2005-06 India Demographic Health Survey data

**DOI:** 10.1186/1471-2458-11-403

**Published:** 2011-05-27

**Authors:** Chandrashekhar T Sreeramareddy, Rahul R Shidhaye, Nalini Sathiakumar

**Affiliations:** 1 Associate Professor, Department of Community Medicine, Melaka-Manipal Medical College, Jalan Batu Hampar, Bukit Baru, Melaka, Malaysia; 2 Lecturer, Indian Institute of Public Health, Vengalrao Nagar, Hyderabad, India; 3 Professor, Department of Epidemiology, School of Public Health, University of Alabama at Birmingham, Birmingham, AL, USA

## Abstract

**Background:**

Observational epidemiological studies and a systematic review have consistently shown an association between maternal exposure to biomass smoke and reduced birth weight. Our aim was to further test this hypothesis.

**Methods**

We analysed the data from 47,139 most recent singleton births during preceding five years of 2005-06 India Demographic Health Survey (DHS). Information about birth weight from child health card and/or mothers' recall) was analysed. Since birth weight was not recorded for nearly 60% of the reported births, maternal self-report of child's size at birth was used as a proxy. Fuel type was classified as high pollution fuels (wood, straw, animal dung, and crop residues kerosene, coal and charcoal), and low pollution fuels (electricity, liquid petroleum gas (LPG), natural gas and biogas). Univariate and multivariable logistic regression models were developed using SURVEYLOGISTIC procedure in SAS system. We used three logistic regression models in which child factors, maternal factors and demographic factors were added step-by-step to the main exposure variable. Adjusted Odds Ratios (AORs) and their 95% CI were calculated. A p-value less than 0.05 was considered as significant.

**Results**

Child's birth weight was available for only 19,270 (41%) births; 3113 from health card and 16,157 from mothers' recall. For available data, mean birth weight was 2846.5 grams (SD = 684.6). Children born in households using high pollution fuels were 73 grams lighter than those born in households using low pollution fuels (mean birth weight 2883.8 grams versus 2810.7 grams, p < 0.001). Use of biomass fuels was associated with size at birth. Unadjusted OR was 1.41 (95% CI, 1.27 1.55). Adjusted OR after controlling for child factors was 1.41 (95% CI 1.29, 1.57). AOR after controlling for both child and maternal factors was 1.21 (95% CI 1.06, 1.32). In final model AOR was 1.07 (95% 0.94, 1.22) after controlling for child, maternal and demographic factors. Gender, birth order, mother's BMI, haemoglobin level and education were significant in all three models.

**Conclusions**

Use of biomass fuels is associated with child size at birth. Future studies should investigate this association using more direct methods for measurement of exposure to smoke emitted from biomass fuels and birth weight.

## Background

Each year about four million neonatal deaths occur worldwide. Nearly 98% of these neonatal deaths occur in developing countries [1]. Globally, one-sixth of all the newborns are low birth weight (LBW, < 2500 grams), which is single most important underlying risk factor for neonatal deaths [1,2]. South Asian countries like India, Pakistan, Bangladesh and Nepal account for nearly half of the LBW babies born in Asia [3]. Despite this figure only about half of the newborns are weighed at birth and for a smaller proportion of them gestational age is known. Therefore, it is difficult to ascertain if LBW is due to intrauterine growth restriction (small-for-gestational age) or prematurity [4]. Studies have shown that maternal smoking and environmental tobacco smoke (ETS) are leading causes for LBW in developed countries [5-7]. However, in developing countries where child mortality rates are higher, factors other than tobacco smoking and ETS have shown to increase risk for LBW [8-10]. Therefore, it is important to identify these risk factors for LBW. Two systematic reviews have reported about the possible association between air pollution and LBW [11,12]. Providing evidence for the association between these risk factors and LBW may form the basis for planning intervention strategies.

Recently, there has been a growing interest about health effects of exposure to biomass smoke [8,13]. Indoor air, which is polluted from burning biomass fuels, may contain carbon monoxide (CO), carbon dioxide (CO2), nitrogen dioxide (NO2), sulphur dioxide (SO2), and volatile organic compounds (VOCs) and particulate material [13-15]. Studies have linked exposure to indoor/biomass smoke with respiratory infections, tuberculosis, cataract, cardiovascular events and also LBW [16]. Smoke emitted from burning biomass fuels contains a large number of air pollutants that have adverse health outcomes [17,18]. In developing countries, a large number of households still depend on biomass fuels for cooking and space heating [13,17]. Biomass fuels are usually burnt on inefficient, unvented household cooking stoves often in poorly ventilated houses or kitchens [9]. Studies from Pakistan [19], Zimbabwe [20], Guatemala [21] and India [22] have reported about possible association of maternal exposure to biomass smoke with reduced birth weight. A study from Guatemala reported that babies born to women who cooked with wood were on an average 63 grams lighter than babies born to mothers who were using either gas or electricity for cooking [21]. Another report based on Zimbabwe Demographic and Health Survey (1999) found that the babies born to mothers who cooked using wood, dung or straw were on average 175 grams lighter than babies born to mothers who cooked with LPG, natural gas or electricity [20]. A retrospective cohort study from Pakistan reported that babies born to mothers who cooked with wood were on average 82 grams lighter than the babies born to mothers who used natural gas [19]. A prospective cohort study from rural south India has reported that exposure to biomass fuel was associated with 49% increased risk of LBW [22].

Though the epidemiological studies mentioned above varied in their designs, they have shown consistent results. A systematic review about the effect of indoor air pollution on LBW has also concluded that the association is consistent despite the limited evidence from epidemiological studies [23]. We aimed to further test the hypothesis that exposure to smoke from burning biomass fuels during pregnancy lowers the birth weight using a large nationally representative sample of households from 2005/6 Demographic Health Survey (DHS) of India. Our objective was to test the association between use of biomass fuels and mother's self-reported size of the child at birth.

## Methods

We conducted a secondary data analysis of the most recent DHS of India. India DHS 2005-06 [also called National Family Health Survey, (NFHS-3)] was carried out under scientific and administrative supervision of International Institute for Population Sciences (IPPS), Mumbai and Macro International during the time period November 2005 to August 2006. The survey collected information about demographic factors, socio-economic factors and health status from a nationally representative probability sample of households. The sample size was 109,041 households selected by two-stage probability proportional to size (PPS) method in rural areas and three stage PPS sampling in urban areas. In both urban and rural areas, primary sampling units were blocks/wards or villages. From each of the chosen sampling units (i.e. village/s or block) random household sampling was done. Within each selected household, all women aged 15 to 49 years were eligible to be respondents for the survey. As primary sampling unit was household, a national household weighing factor was used to maximise representativeness of the sample.

Data were collected according to a standard protocol. Three core survey questionnaires i.e. the Household Questionnaire, the Woman's Questionnaire and the Man's Questionnaire were translated into 18 local languages and field tested. Subsequently, filled questionnaires were back-translated to English. These questionnaires were used in all 29 states of India. Questionnaires used in each state were bilingual, with questions in both principal languages of the state and English. To minimize language barriers, the survey was administered by trained interviewers either in English or principal language of the state or preferred language of the household. Further details of sampling design, training of survey team, survey management and quality control measures are separately documented in the country reports published by ORC (Opinion Research Corp.) Macro International [24]. From a sample of 109,041 households a total of 124, 385 female participants were interviewed giving a response rate of 94.5%. For our analyses, the respondents were ever married women aged between 15 to 49 years who had given birth to at least one child. All these participants were asked about births during five years prior to the survey (interview) date. For each birth, details about place of birth, birth weight, gender and size of the baby at birth were asked.

### Ethical considerations and consent

The Independent Review Boards (IRB) of IPPS and ORC Macro international had independently reviewed the DHS protocols, data collection tools and procedures and provided ethical approval. Approval was also taken from the IRBs of Melaka-Manipal Medical College, Malaysia and School of Public health, University of Alabama at Birmingham. The interviewers informed the participants that participation in the survey was voluntary and assured them that information provided will be kept confidential. Following this an informed consent was obtained from each participant.

### Outcome variable

The main outcome measure used was child's birth weight. For each birth during the preceding five years, mothers were asked about birth weight. Mothers were asked, "Was your baby weighed at birth?" If the response was 'yes', they were further questioned, "How much did your baby weigh?" If mother possessed a health card, she was asked to show it and birth weight was recorded. For those mothers who did not possess a health card, birth weight was recorded according to mother's recall. Mothers were also asked to classify 'size of the baby' at birth into of following five categories: 'very large', 'larger than average', 'average',' smaller than average', and 'very small'. We pooled these categories to form a binary variable as 'greater than or equal to average size' and 'less than average size' at birth corresponding to normal birth weight (≥2500 grams) and low birth weight (birth weight < 2500 grams) respectively. Births with missing information about size at birth and multiple births (6.7%) were excluded from our analysis. In a similar study based on Zimbabwe DHS, the authors have acknowledged about bias arising from a disproportionately high number of babies from households using biomass fuels being not weighed at birth as compared to the households using cleaner fuel [20]. We observed a similar pattern in our data also. Nearly 60% of the births included for our analysis did not have information about birth weight either from mother's recall or health card, whereas for 'size at birth' missing information was only 4.5%. This precluded us from using information about birth weight as outcome variable) from health card and/or mother's recall. Therefore size at birth was used as a proxy for birth weight to classify children as low birth weight and normal birth weight. This gave us a bigger sample size of 47,139 births to be included in the final analysis

### Exposure variables

The standard DHS used eleven fold classification of cooking fuels used in the house. The specific question asked was, "What type of fuel does your household mainly use?" For our analysis, the main cooking fuel used was grouped into one of the two categories. These two categories were namely; high pollution fuels (wood, straw, animal dung, and crop residues, kerosene, coal and charcoal), and low pollution fuels (electricity, liquid petroleum gas, natural gas and biogas).

### Other predictor variables

The DHS also collected information about smoking habits of the individuals in households, and tested haemoglobin level for women. Mothers were asked, "Do you currently smoke cigarettes?" The effect of exposure to biomass fuels on birth weight may be confounded by factors like tobacco smoking and nutritional status of mother. Therefore, we adjusted for body mass index (BMI) as an indicator for nutritional status of mothers [25] and haemoglobin level of mothers along with biological and socio-demographic factors. Blood haemoglobin level, height and weight measurements were obtained by the interviewer at the time of interview. Blood haemoglobin level was measured using a portable HemoCue system which is feasible to operate in field setting. HemoCue system uses a drop of blood taken by finger prick. Blood was drawn into a cuvette and inserted into a portable, battery-operated instrument which gives a digital reading of haemoglobin concentration. Haemoglobin measurements were adjusted for altitude. The respondents were informed about the results of haemoglobin testing. As an indicator of nutritional status, maternal BMI was calculated using height and weight. During the interview, maternal demographic information about age at interview, age at the time of each birth, gender of the child, birth order, highest level of education attained, religion practiced, rural or urban area of residence, and level of household wealth was also obtained. Maternal education was classified as no formal education, primary education, secondary education, or higher education. Mother's religion was classified as Hindu, Muslim, Christian, Buddhist/Neo-Buddhist, or other. Urban or rural area of residence was defined as a mega city, large city, small city, large town, small town, or rural area. Wealth Index which is a relative index of household wealth was calculated based on a standard set of household assets as observed by the interviewer, ownership of consumer items and dwelling characteristics. The individuals were ranked on the basis of their household score and divided into quintiles where one is the poorest 20% of the households and five is the wealthiest 20% of the households.

### Statistical analyses

We did an exploratory analysis of birth weight of the child obtained from health card and mother's recall. We compared the mean birth weight between two fuel type categories i.e. high pollution and low pollution fuels using independent samples t-test. To test the concordance between birth weight of child (from recall and health card) and size at birth we used Somer's D test. The outcome variable was size at birth dichotomized into normal birth weight and low birth weight. For bivariate analysis of categorical variables we used chi square test. We carried out the analyses using SAS software (Cray, North Carolina). In addition to type of fuel used, other (independent) variables considered were child factors (gender, and birth order), maternal factors (anaemia, BMI, age at childbirth, smoking, education) and socio-demographic factors (religion, wealth index, urban/rural residence). We tested for the possibility of collinearity between predictor variables. In a correlation matrix, BMI versus haemoglobin level and wealth index versus type of cooking fuel were paired and Pearson's correlation coefficients were calculated to rule out multicollinearity. Multilevel modelling was done to adjust for cluster sampling (cluster as primary sampling unit used in DHS). Three multivariable regression analysis models were built using SURVEYLOGISTIC procedure in SAS system (version 8.02) [26]. During statistical modelling, certain factors known to confound the results were adjusted. Adjusted odds ratios (AOR) and their 95% confidence intervals (95% CI) were calculated. A p-value less than 0.05 was considered as significant.

## Results

### Sample distribution

A total of 124, 385 women participants reported 56, 438 births during the previous five years. The final sample analysed was 47,139 most recent singleton births. The main types of fuel used by the households were as follows: electricity (267, 0.6%), liquid petroleum gas (12333, 26.2%), biogas(174, 0.4%), kerosene (1708, 3.6%), coal & lignite (786, 1.7%), charcoal (286, 0.6%), wood (24049, 51%), straw/shrubs/grass (1807, 3.8%), agricultural crop ( 1226, 2.6%), and animal dung ( 4503, 9.6%). Birth weight of the child was available for only 19,270 (41%) births. Of these, birth weights for 3113 children were available from child health card (18.4% were LBW) and for 16,157 children from mother's recall (20.5% were LBW). Overall mean birth weight was 2846.5 grams (SD = 684.6). The children born in households using high pollution fuels were 73 grams lighter than the children born in households using low pollution fuels (mean birth weights; 2883.8 grams versus 2810.7 grams). This difference was statistically significant (p < 0.001). The adjusted (for wealth index) mean difference was 39.9 grams (SD = 13.4).

The distribution of birth weights of most recent births according to type of fuel used in the households, mothers' education, and type of residence is shown in Table 1. For 6.6% of the babies, birth weight was available from health card and for 34.3% of the babies; birth weight was recorded from mother's recall. Babies who were not weighed at birth were more likely to be from households using high pollution fuels as compared to households using low pollution fuels (66.87% versus 18.81%). A similar pattern was observed according to the type of residence, i.e. rural versus urban: 68.1% versus 31.3%. Additionally, babies who were not weighed at birth were more likely to be born to mothers who had lesser education. Mothers had a tendency of reporting the size at birth as 'average' since about 57% of the babies was reported as 'average size' (table 2). The distribution of five categories of size at birth between households using high pollution fuels and low pollution fuels was statistically significant (p < 0.001).

**Table 1:** Distribution of sample of birth weights (from recall and card) according to main exposure variable and selected socio-demographic factors.

Variable	Weighed at birth		Not weighed at birth	Do not know	Total births
				
	From card	From recall			
**Type of fuel**					

High pollution	1441 (66.9)	8387 (4.2)	22981(24.4)	1556 (4.5)	34365 (100)

Low Pollution	1672 (19.3)	7779 (13.1)	2404 (60.9)	859 (6.7)	12774 (100)

**Educational status**					

No education	340 (1.7)	2798 (14.4)	15445 (79.5)	948(4.4)	19431 (100)

Primary	298 (4.4)	2117 (31.2)	3965 (58.5)	396 (5.8)	6776 (100)

Secondary	1858 (10.7)	8721(50.2)	5790 (33.3)	1008 (5.8)	17377 (100)

Higher	617 (17.4)	2530 (71.2)	244 (6.9)	163 (4.6)	3554 (100)

**Type of residence**					

Urban	1958 (10.9)	9203 (51.0)	5647 (31.3)	1288 (6.9)	18046 (100)

Rural	1155 (4.0)	6963 (23.9)	19798 (68.1)	1177 (4.0)	29093 (100)

**Total births**	3113 (6.6)	16166 (34.3)	25444 (54.0)	2415 (5.1)	47139 (100)

* Figures in parenthesis are row percentages

**Table 2:** Distribution of size at birth according to type of fuels for births during five years of prior to the survey.

Size at birth	Type of fuel		Total (%)
		
	High pollution	Low Pollution	
Very large	1181(3.44)	590 (4.62)	1771 (3.76)

Larger than average	6280 (18.27)	2676 (20.95)	8956 (19.0)

Average	19270 (56.07)	7492 (58.65)	26762 (56.77)

Smaller than average	5288 (15.38)	1466 (11.47)	6754 (14.33)

Very small	2346 (6.82)	550 (4.30)	2896 (6.14)

**Total**	34365 (100)	12774 (100)	47139 (100)

Chi square = 276.35 degrees of freedom = 4, p = < 0.001

We used the test of association (Somer's D) for concordance between birth weight of child (from mother's recall and health card) and size at birth (table 3). Out of 3889 babies who were reported as weighing less than 2500 grams, 2066 (53.1%) were perceived by mothers as less than average size at birth. Similarly, out of 15,389 babies who were reported as weighing 2500 grams or more, 13,990 (90.9%) babies were perceived by mothers as average or more than average size at birth. These numbers suggest that mother's perception about size at birth was reasonably reliable.

**Table 3:** Test of concordance between birth weight of child (from recall and health card) and size at birth

	Birth weight of the child		Total
		
	< 2500 grams	≥ 2500 grams	
Greater than or equal to average	1823 (46.9)	13990 (90.9)	15813

Less than average	2066 (53.1)	1388 (9.1)	3454

Total	3889	15389	19267

### Univariate analysis

The results of univariate analysis for type of fuel and other control variables with low birth weight i.e. the effect of cooking fuel, tobacco smoke, biological factors and other variables on low birth weight are shown in table 4. Mothers from households using high pollution fuels were 1.4 (OR 1.41, 95% CI 1.27, 1.55) times more likely to give birth to a low birth weight baby as compared to those using cleaner fuels. Mothers who reported to be smoking were 1.4 times (OR 1.43, 95% CI 1.11, 1.86) more likely to give birth to a LBW baby as compared to those who did not reported to be smoking during the time of survey. Female babies (OR 1.13, 95% CI 1.07, 1.20) and babies of higher birth order (OR 0.89, 95% CI 0.83, 0.96) were less likely to be LBW. Among maternal factors, lower haemoglobin level, lower educational status, and lower BMI increased the risk for child being LBW. Similarly children born in rural areas and those belonging to families with lower wealth index were at increased risk of being LBW.

**Table 4:** Univariate analyses of size at birth with type of fuel and other variables

Variable	Low birth weight (n = 9650)	Normal birth weight (n = 37489)	Unadjusted Odds ratios (95% CI)	p-value
**Type of fuel**				

Low Pollution Fuels	2016	10758	1	

High Pollution Fuels	7634	26731	1.41 (1.27, 1.55)	< 0.0001

**Gender of the baby**				

Male	4763	19741	1	

Female	4887	17748	1.13 (1.07, 1.20)	< 0.0001

**Birth order**				

1	3166	11437	1	

2	2532	10581	0.89 (0.83, 0.96)	0.014

3	1527	6233	0.94 (0.85, 1.03)	0.592

≥ 4	2485	9238	0.97 (0.89, 1.06)	0.390

**Mother's age at child birth**				

≤ 24	8194	31490	1	

25-34	988	4499	0.90 (0.80, 1.01)	0.711

35-49	33	126	0.94 (0.46, 1.94 )	0.980

**Wealth Index**				

Poorest	2080	6345	1	

Poorer	2023	6679	1,60 (1.42, 1.81)	<0.0001

Middle	2095	7358	1.51 (1.34, 1.70)	< 0.0001

Richer	1929	8485	1.41 (1.25, 1.58)	< 0.0001

Richest	1523	8442	1.18 (1.05, 1.32)	0.0011

**Type of residence**				

Urban	3170	14876	1	

Rural	6480	22613	1.22 (1.11, 1.34)	< 0.0001

**Mother's religion**				

Hindu	6763	25540	1	

Muslim	1606	6247	0.98 (0.88, 1.1)	0.748

Christian	770	4029	0.76 (0.63, 0.92)	0.011

Others	369	1242	1.18 (0.97, 1.42)	0.059

**Mother's Body Mass Index**				

≤18	2724	8723		

18-23	5298	20209	0.87 (0.82, 0.94)	0.0006

23-28	1055	5577	0.64 (0.57, 0.72)	0.0001

28-40	245	1363	0.63 (0.50, 0.80)	0.0170

Maternal haemoglobin level (adjusted for altitude) in mg/dl *				

	137.8	148.5	0.998 (0.997,0.999)	0.001

**Mother's education**				

No education	4468	14963	1	

Primary	1491	5285	0.96 (0.88, 1.07)	0.094

Secondary	3244	14133	0.83 (0.76, 0.89)	< 0.0001

Higher	447	3554	0.55 (0.46, 0.65)	< 0.0001

**Maternal smoking**				

No	9455	36937	1	

Yes	192	536	1.43 (1.11, 1.86)	0.0067

* 44482 women had undergone haemoglobin testing. Median = 117 (Q1=105, Q3=128)

### Multivariable analysis

We checked for collinearity between BMI and maternal haemoglobin level and there was no correlation (r = 0.04). A strong collinearity was present (r = 0.664) between wealth index and type of cooking fuel. Table 5 shows the results of the multivariable models. We developed three models by including child factors (model-1), maternal factors (model-2) and socio-demographic factors (model-3), one by one to the main exposure variable i.e. type of cooking fuel, to control for the effect of these variables on the association between exposure to biomass fuel smoke and birth weight. In model-1, when gender of the baby and birth order were statistically controlled, the adjusted OR for the association i.e. effect of cooking fuel on birth weight was the highest (adjusted OR 1.41; 95% CI 1.29, 1.57). In model-1, the female gender has a protective effect for LBW or females babies tended to have a higher birth weight and similarly higher birth order showed a protective effect for LBW i.e. higher birth order babies had a higher birth weight. In model-2, when maternal characteristics like age at child birth, maternal smoking, educational status, BMI and haemoglobin were added, the effect of cooking fuel on birth weight remained significant but effect size decreased (adjusted OR 1.21 95% CI 1.06, 1.32). This decreased effect may be have been due to positive effect shown by BMI (p = 0.0065) and haemoglobin level (p = 0.0019) for birth weight. Further, the effect of cooking fuel on birth weight may have been confounded by maternal education which was protective for LBW. The reason for this could be that women of higher educational status belonged to households using cleaner fuels. Baby girls were likely to have higher birth weight and higher birth order was positively associated with birth weight, in model-2 as well. Maternal smoking and mother's age at child birth were not statistically significant. In full model (model-3) in addition to child and maternal characteristics, demographic variables like religion, wealth index, type of residence (urban/rural) were also statistically controlled. In model-3, there was no association (p = 0.26) between cooking with biomass fuels and birth weight (adjusted OR 1.07, 95% 0.94, 1.22). The type of residence was not associated with birth weight (p = 0.68). However, wealth index had a positive association with birth weight i.e. higher wealth index had higher birth weight (p = 0.0011, adjusted OR 0.93 95% CI 0.89, 0.97). Wealth index might have masked the association between cooking fuel and birth weight in model-3. This may imply that people with higher income could afford to buy cleaner fuels which are usually more expensive. The effect of child's gender, birth order, mother's education, BMI, and haemoglobin level on birth weight remained significant. Age at child birth and maternal smoking which was not significant in models one and two were not significant in the final model as well.

**Table 5:** Multivariable analysis of size at birth with type of fuel and other variables

Variable	Model 1	Model 2	Model 3
**Type of fuel**			

Low Pollution Fuels	1	1	1

High Pollution Fuels	1.41 (1.29, 1.57) ***	1.21 (1.06, 1.32) **	1.07 (0.94, 1.22)

**Gender of the baby**			

Male	1	1	1

Female	0.88 (0.83, 0.94) ***	0.88 (0.83, 0.94) ***	0.88 (0.83, 0.93) ***

**Birth order**			

1	1	1	1

2	0.88 (0.82, 0.95) **	0.88 (0.81, 0.95) **	0.87 (0.81, 0.94) **

3	0.89 (0.82, 0.98) **	0.86 (0.78, 0.94) **	0.85 (0.77, 0.94) **

≥ 4	0.91 (0.83, 0.99) **	0.83 (0.75, 0.92) **	0.81 (0.74, 0.89) **

**Mother's age at childbirth**			

≤ 24		1	1

25-34		1.08 (0.95, 1.22)	1.08 (0.95, 1.23)

35-49		1.15 (0.52, 2.55)	1.16 (0.52, 2.59)

**Mother's Body Mass Index**			

≤ 18		1	1

18-23		0.92 (0.85, 0.99) ***	0.93 (0.86, 1.01)

23-28		0.74 (0.66, 0.84) **	0.77 (0.68, 0.87) *

28-40		0.77 (0.61, 0.97) **	0.79 (0.63, 1.01)

**Mother's education**			

No education		1	1

Primary		0.96 (0.87, 1.07)	0.99 (0.89, 1.10)

Secondary		0.86 (0.78, 0. 94) *	0.92 (0.83, 1.02)

Higher		0.62 (0.51, 0.76) *	0.67 (0.54, 0.82) **

**Mother smokes**			

No		1	1

Yes		1.25 (0.94, 1.66)	1.24 (0.93, 1.65)

**Mothers haemoglobin level (adjusted for altitude) in mg/dl §**			

		1	1

		0.99 (0.99, 0.99) **	0.99 (0.99, 0.99) **

**Wealth Index**			

Poorest to richest			1

			0.93 (0.89, 0.97) **

**Type of residence**			

Urban			1

Rural			0.97 (0.87, 1.09)

**Religion**			

Hindu			1

Muslim			0.94 (0.83, 1.05)

Christian			0.81 (0.63, 1.04)

Others			1.38 (1.12, 1.72) *

§ beta coefficient = -0.00134, standard error = 0.000428* p < 0.05, ** p < 0.01, *** p < 0.001

## Discussion

The results from our analysis suggest that use of biomass cooking fuels is associated with child's birth weight and size at birth. The children born in households using high pollution fuels had significantly lower birth weight than those children born in households using low pollution fuels. The association between use of high pollution fuels and size at birth was independent of child's gender, birth order, mother's education and nutritional status, even after adjustment. The association did not remain significant after adding socio-economic factors in the final model.

The results of our analysis about the association between type of cooking fuel and LBW are consistent with previous reports from rural Guatemala, Pakistan, Zimbabwe and Southern India [19-22]. The only difference of our results from previous studies is the possibility of residual confounding effect of household wealth index. The effect of type of fuel on LBW may have been masked by wealth index in the final regression model. Wealth index being a composite measure of cumulative living standard is computed based on 41 items including type of fuel used in households. Therefore we did not find any association in the final regression model. One reason for such an effect may be due to correlation between wealth index and type of fuel (r = 0.664), urban/rural residence and type of fuel used (r = 0.54), which could not be controlled for in the modelling. However, the influence of other variables well known to cause LBW remained significant during multivariable modelling. These consistent associations of primary exposure variable and the covariates should be discussed in the light of various epidemiological study designs and other criteria necessary to establish causal links. LBW was associated with maternal smoking in studies from Southern India and Pakistan [19,22]; mother's age in studies from Pakistan and Guatemala [19,21]; birth order/parity in studies from Guatemala and Zimbabwe [20,21], and BMI in studies from Zimbabwe and Pakistan [19,20]. Though all the variables were not tested in all the studies and the degree of adjustment varied across studies remarkably, the association has remained consistent. In our analysis, birth weight was associated urban/rural residence and literacy. Such associations may be due interactions between socio-economic factors as discussed above.

The study from Guatemala used a non-representative sample with predominantly hospital deliveries [21]. The study from Pakistan had a population-based historical cohort allowing to test for temporal sequence i.e. use of wood fuel preceding the birth of LBW baby [19]. The report from Zimbabwe used birth weight information from recall and health card and had selection bias as mentioned earlier i.e. birth weights were not available for babies from a disproportionately high number of households using high pollution fuels [20]. In Zimbabwean study, selection bias arising from these differentials in birth weight information may have lead to underestimation of actual effect size for the association. Maternal self-reports of birth weight during health interviews are not accurate and have a propensity for over-estimation [27]. When a sensitivity analysis was carried out overestimation of birth weight had lead to attenuation of effect size in the study from Zimbabwe [23]. The study from Southern India used a cohort design and did not adjust for nutritional factors in their analysis but tested the exposure to second hand tobacco smoke (SHTS) [22]. However, very few of the earlier studies have used direct methods for measurement of indoor air pollution from burning biomass fuels. Evidence for association between ETS and LBW [6,7] from the systematic reviews and similarity of pollutants in ETS and biomass smoke [8,17] is a sufficient proof for the hypothesis tested in our study. Studies measuring ambient air pollutants like sulphur dioxide, carbon monoxide and PM_10 _have also shown that these pollutants are associated with decreased birth weight [28-30]. The association is also supported by biological plausibility from animal studies. It has been has shown that when rabbits were exposed to 90 ppm of CO, 8-9% of COHb was produced and there was 11% reduction in mean birth weight [31]. Studies have also demonstrated that air pollutants can bring about changes in alveolar macrophage load [18], cross the alveolar-capillary barrier and penetrate deep into the lungs [32].

Like any other epidemiological study, our study has certain strengths and limitations. The strength of our analysis was using a large nationally representative sample. Additionally, using size at birth as proxy for birth weight gave us a bigger sample and increase in statistical power for our results. We examined the effect of maternal smoking, maternal BMI and haemoglobin levels on size at birth. However, our results should be interpreted with caution against the inherent limitations of cross-sectional design and secondary data analysis. The main limitations are lack of temporality of association due to cross-sectional design, and possible misclassification of both exposure and outcome assessment. Due to cross-sectional design of DHS, it was not possible to ascertain if exposure (type of fuel use) occurred prior to the outcome (size of baby at birth) or vice versa. Another main limitation was misclassification bias of both exposure and outcome. For the information about type of fuel used, we relied on one question in the DHS questionnaire. Information about multiple fuel use, temporal changes (time trend) in fuel use, duration of fuel used were not assessed leading to exposure misclassification. Type of fuel used during pregnancy may have been different from that being used at the time of survey. The main outcome i.e. birth weight was not available for majority of births. The use of size of baby at birth as proxy to birth weight has lead to misclassification bias. Mothers were generally very good at identifying non-LBW babies. Only about half of the babies whose birth weight was reported or recorded as below 2500 grams were correctly classified as less than average size at birth (i.e. LBW). This low sensitivity of outcome measure may have lead to attenuation of association (effect size) in our final multivariable analysis. The use of actual birth weight as recorded from child health card and/or mother's recall as outcome measure would have led to selection bias (a large proportion of babies were not weighed in high pollution fuels compared to low pollution fuels i.e. 71% versus. 26.1%). Similarly, women from more affluent households, with better education were likely to have recorded birth weight. Use of birth weight as recorded from child health card may also lead to misclassification bias. This is because birth weight recording in developing country like India is usually imprecise except for hospital births. In India, where are high proportion of births are taking place at home, weight is measured at a convenient time. We did not have information about some important determinants of birth weight from the DHS data i.e. foods consumed and physical activity, and exposure to second hand smoke during pregnancy. We could not examine the effect of these variables on size at birth in multivariable analysis.

We could not assign the households using combination of fuels into a different category since we did not have such information from DHS data. We lacked information about duration of exposure, and stage of pregnancy when exposure had occurred. The women exposed to biomass smoke may have had higher rates of still birth [23] and miscarriages. Since we included only live births, the proportion of pregnancies that may have lead to LBW babies is not known. Our effect size might have been an underestimate due to the reasons mentioned above. The data on birth weight was collected for previous five years and during this period the households might have switched from high pollution (biomass) fuels to cleaner fuels for cooking. We did not have the data about this to carry out a separate analysis. The birth weight and size at birth we analysed were for most recent births during five years prior to the date of survey (index pregnancy). Some of the confounding variables we used in our multivariable analysis i.e. haemoglobin, BMI, smoking were done during the time of survey. However, these measurements may not have been the same during the index pregnancy to which they were applied. Therefore, our results should be interpreted with caution.

Despite these limitations, the consistency of the association from various studies and systematic reviews [23] provides a strong case for public health policy and programs to reduce exposure to smoke from biomass fuel. As exposure to smoke from biomass fuel is known to cause other health effects [16,33,34] as well, public information campaigns about health risks of exposure should be given. In areas where shifting to cleaner fuels is not feasible due to financial reasons, well designed stoves to improve combustion and ventilation by use of chimneys (an outlet for smoke) should be promoted.

## Conclusion

Use of biomass fuels is associated with child size at birth. Our results are consistent with previous observational studies in different developing country settings. Future studies should investigate this association using robust study designs which use more direct methods for measuring level of exposure to smoke emitted from burning biomass fuels and clinical parameters like birth weight and other pregnancy outcomes.

## Competing interests

The authors declare that they have no competing interests.

## Authors' contributions

CTS conceptualised the study, wrote the project proposal and write the first draft of manuscript. RRS assisted in conceptualisation, carried out data analysis, interpretation of results and commented on draft manuscript. NS assisted in conceptualisation, assisted in data analysis and commented on draft manuscript. All the authors read and approved the final manuscript.

## Pre-publication history

The pre-publication history for this paper can be accessed here:

http://www.biomedcentral.com/1471-2458/11/403/prepub
